# Differential Lyn-dependence of the SHIP1-deficient mast cell phenotype

**DOI:** 10.1186/s12964-016-0135-0

**Published:** 2016-05-20

**Authors:** Susana M. Nunes de Miranda, Thomas Wilhelm, Michael Huber, Carolin N. Zorn

**Affiliations:** Institute of Biochemistry and Molecular Immunology, University Clinic, RWTH Aachen University, Pauwelsstraße 30, Aachen, 52074 Germany

**Keywords:** Lyn, Mast cell, SH2-containing inositol-5’-phosphatase, Src family kinase

## Abstract

**Background:**

Antigen (Ag)/IgE-mediated mast cell (MC) responses play detrimental roles in allergic diseases. MC activation via the high-affinity receptor for IgE (FcεRI) is controlled by the Src family kinase Lyn. Lyn-deficient (-/-) bone marrow-derived MCs (BMMCs) have been shown by various laboratories to exert stronger activation of the PI3K pathway, degranulation, and production of pro-inflammatory cytokines compared to wild-type (wt) cells. This mimics the phenotype of BMMCs deficient for the SH2-containing inositol-5’-phosphatase 1 (SHIP1). In this line, Lyn has been demonstrated to tyrosine-phosphorylate and activate SHIP1, thereby constituting a negative feedback control of PI3K-mediated signals. However, several groups have also reported on Lyn-/- BMMCs degranulating weaker than wt BMMCs.

**Results:**

Lyn-/- BMMCs, which show a suppressed degranulation response, were found to exhibit abrogated tyrosine phosphorylation of SHIP1 as well. This indicated that even in the presence of reduced SHIP1 function MC degranulation is dependent on Lyn function. In contrast to the reduced immediate secretory response, pro-inflammatory cytokine production was augmented in Lyn-/- BMMCs. For closer analysis, Lyn/SHIP1-double-deficient (dko) BMMCs were generated. In support of the dominance of Lyn deficiency, dko BMMCs degranulated significantly weaker than SHIP1-/- BMMCs. This coincided with reduced LAT1 and PLC-γ1 phosphorylation as well as Ca^2+^ mobilization in those cells. Interestingly, activation of the NFκB pathway followed the same pattern as measured by IκBα phosphorylation/degradation as well as induction of NFκB target genes. This suggested that Ag-triggered NFκB activation involves a Ca^2+^-dependent step. Indeed, IκBα phosphorylation/degradation and NFκB target gene induction were controlled by the Ca^2+^-dependent phosphatase calcineurin.

**Conclusions:**

Lyn deficiency is dominant over SHIP1 deficiency in MCs with respect to Ag-triggered degranulation and preceding signaling events. Moreover, the NFκB pathway and respective targets are activated in a Lyn- and Ca^2+^-dependent manner, reinforcing the importance of Lyn for MC activation.

**Electronic supplementary material:**

The online version of this article (doi:10.1186/s12964-016-0135-0) contains supplementary material, which is available to authorized users.

## Plain English summary

Mast cells play a detrimental role in allergic diseases, such as rhinitis and asthma. Allergens are recognized by specific receptors on the mast cell’s surface (so-called IgE-bound FcεRIs) and the resulting allergen-triggered activation of the mast cell results in pro-inflammatory responses like degranulation and cytokine secretion. These responses are controlled by intracellular signaling proteins, which functionally connect the allergen-binding receptor at the cell surface with the execution of the respective pro-inflammatory response. Whereas degranulation means an immediate release of preformed mediators, such as histamine and proteases, cytokine production and secretion is an elaborate prolonged process involving gene transcription, protein production, and protein secretion. Amongst the many signaling proteins controlling such activation processes are so-called positive and negative regulators, which together shape the physiological extent and kinetic of the cell’s reaction. In the present work, the function and interplay of a dominant negative regulator (called SHIP1) and another regulator (called Lyn), which exhibits both positive and negative functions, are analyzed in murine mast cells. We show that SHIP1 and Lyn are negatively impacting on allergen-triggered cytokine production, whereas these proteins have opposing functions with respect to the regulation of degranulation. While SHIP1 is a negative regulator of this response, Lyn is important for its activation. Thus, these results offer an instructive example for the context- and time-dependent function of signaling proteins, in particular Lyn.

## Background

Signaling from the high-affinity receptor for IgE (FcεRI) is dependent on the Src family kinase Lyn, which is pre-associated with the FcεRI β-chain [1, 2] and phosphorylates the ITAMs of the FcεRI β- and γ-chains upon Ag-triggered activation [3]. Analyses of Lyn-deficient (-/-) mast cells (MCs), in particular bone marrow-derived MCs (BMMCs), revealed antithetic roles of Lyn in regulating FcεRI-mediated MC functions. Lyn activity strongly depended on the extent and quality of FcεRI crosslinking. Xiao et al. elegantly demonstrated that in situations of weak FcεRI crosslinking (e.g. stimulation of MCs with cytokinergic IgE or IgE-loaded MCs with anti-IgE antibodies) Lyn acts as a positive regulator [4]. Consequently, in response to such stimuli Lyn-/- MCs showed reduced activation compared to wild-type (wt) cells [4]. Contrariwise, in response to stimuli strongly crosslinking the FcεRI (e.g. stimulation of anti-DNP IgE-loaded MCs with DNP_30-40_-HSA) Lyn was shown to be a negative regulator of MC activation. Thus, compared to wt cells, Lyn-/- BMMCs exerted augmented responses to strongly crosslinking Ag [4, 5]. In addition, Lyn function appeared to be dependent on the genetic background of the respective mice/BMMCs. Yamashita et al. reported on a clear-cut difference in degranulation between Lyn-deficient BMMCs of C57BL/6 and 129/Sv genetic background with C57BL/6 Lyn-/- BMMCs degranulating less and 129/Sv Lyn-/- BMMCs degranulating stronger than the respective wt cells [6]. However, other groups have reported on augmented degranulation of C57BL/6 Lyn-/- BMMCs compared to wt BMMCs [4, 5, 7, 8], suggesting that not only genetic background, but also culture conditions might contribute to phenotypic manifestations. Interestingly, irrespective of the genetic background of BMMCs studied (C57BL/6, 129/Sv or mixed background thereof) and the resulting degranulation phenotype, Ag-triggered Lyn-/- BMMCs showed reduced overall substrate tyrosine phosphorylation (Y-P) and Ca^2+^ mobilization as well as enhanced pro-inflammatory cytokine secretion compared to wt BMMCs [4–9].

In the study by Hernandez-Hansen et al., augmented Ag-induced degranulation, especially at high (= supra-optimal) Ag concentrations, resembled the Ag dose-response found in BMMCs deficient for the SH2-containing inositol-5’-phosphatase SHIP1 [5, 10, 11]. In correlation, Lyn deficiency was connected to reduced Ag-triggered SHIP1 Y-P and activity resulting in enhanced phosphorylation of PKB (a.k.a. Akt) [4, 5]. So far, such functional interaction between Lyn and SHIP1 was only investigated in BMMCs, in which the degranulation phenotype of Lyn- and SHIP1-deficient MCs concurred. Thus, in Lyn-/- BMMCs showing reduced degranulation compared to the respective wt cells, functional inter-dependence between Lyn and SHIP1 has not been clarified yet.

In this study, we showed that even in Lyn-/- BMMCs, which degranulated less than the respective wt cells, Ag-triggered SHIP1 Y-P was strongly diminished compared to wt cells. This suggested that Lyn deficiency is able to overcome a signaling situation of reduced SHIP1 phosphorylation/activation, which, theoretically, should be characterized by enhanced degranulation. Analyzing Lyn/SHIP1 dko BMMCs, we demonstrated that Lyn deficiency is, indeed, dominant over SHIP1 deficiency with reduced degranulation, Ca^2+^ mobilization, and relevant signal transduction events in dko BMMCs compared to SHIP1-/- cells. Intriguingly, our studies also showed Lyn and Ca^2+^/calcineurin dependence of FcεRI-mediated activation of the NFκB pathway. Moreover, Lyn-/-, SHIP1-/-, and dko BMMCs resembled each other with respect to production and secretion of pro-inflammatory cytokines. In summary, Lyn deficiency concurs with SHIP1 deficiency with respect to Ag-triggered PKB phosphorylation and cytokine production, however it is dominant over the absence of SHIP1 with regard to Ca^2+^ mobilization, degranulation, and relevant signaling.

## Results

### Reduced Ag-triggered degranulation in Lyn-deficient mast cells despite suppressed tyrosine phosphorylation of SHIP1

In hematopoietic cells, the lipid phosphatase SHIP1 is a crucial negative regulator of PI3K-mediated processes and compared to wt BMMCs, SHIP1-deficient (-/-) BMMCs showed augmented Ca^2+^ mobilization, degranulation, PKB activation, and pro-inflammatory cytokine production in response to IgE/Ag stimulation [10, 12, 13]. SHIP1 phosphatase activity was connected to Lyn-mediated Y-P of SHIP1 [5]. Thus, it was not unexpected that Lyn deficiency resulted in enhanced Ag-triggered degranulation, PKB phosphorylation, and cytokine production as well [4–8]. However, Lyn-/- BMMCs have also been reported repeatedly to degranulate less compared to respective wt cells [6, 9, 14], which does not accord with the reported functional interaction between Lyn and SHIP1. So far, the functional connection between Lyn and SHIP1 was not addressed in such Lyn-deficient BMMCs that showed reduced degranulation compared to wt cells.

**Fig. 1:**
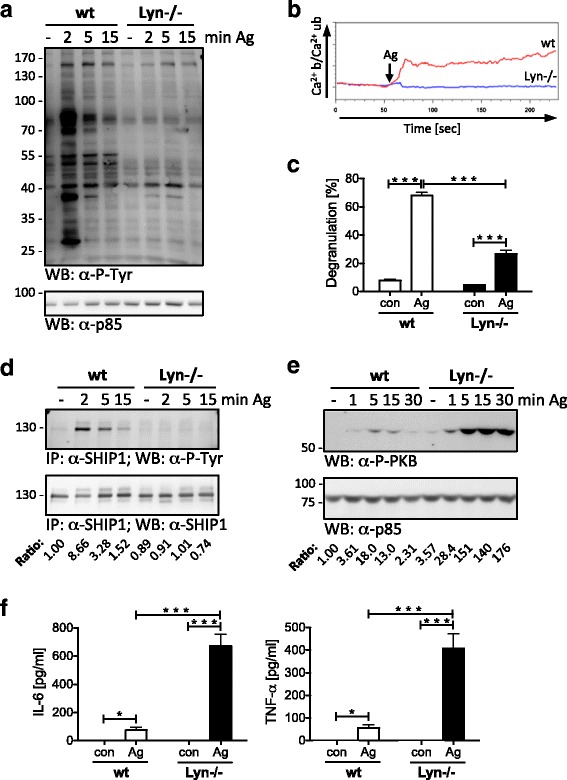
Reduced degranulation of Lyn-/- BMMCs despite abolished SHIP1 tyrosine phosphorylation. Wt and Lyn-/- BMMCs were preloaded and starved overnight with 0.15 μg/ml IgE. **a** BMMCs were left untreated (-) or stimulated with 200 ng/ml Ag (DNP-HSA) for the indicated time points. Whole-cell lysates were subjected to WB analysis with antibodies against phospho-tyrosine (P-Tyr; upper panel) and p85 (loading control, lower panel). Comparable results were obtained with 20 ng/ml Ag (see Fig. 3c). **b** Ca^2+^ mobilization was measured in wt and Lyn-/- BMMCs for 4 min by flow cytometry using the Ca^2+^-sensitive fluorescent dyes fluo-3 (Ca^2+^ b (bound)) and fura red (Ca^2+^ ub (unbound)). Steady-state fluorescence was measured for 1 min before BMMCs were stimulated with Ag (20 ng/ml). The arrow marks the time point of stimulus addition. For other Ag concentrations see Additional file 1: Figure S1. **c** Unstarved BMMCs were left untreated (con) or stimulated with Ag (20 ng/ml) for 20 min. Degranulation was determined by β-hexosaminidase assay. Each point is the mean of triplicates ± SD. **d** BMMCs were left unstimulated (-) or stimulated with Ag (200 ng/ml) for the indicated time points. Whole-cell lysates were subjected to anti-SHIP1 immunoprecipitation (IP) and WB analysis was performed with antibodies against P-Tyr (upper panel) and SHIP1 (lower panel). **e** BMMCs were left unstimulated (-) or stimulated with Ag (20 ng/ml) for the indicated time points. Whole-cell lysates were subjected to WB analysis with antibodies against P-PKB (upper panel) and p85 (loading control, lower panel). Densitometry was performed and relative expression levels are indicated under each band. Comparable results were obtained with 200 ng/ml Ag (see Fig. 2e). **f** BMMCs were left unstimulated (con) or stimulated with Ag (20 ng/ml) for 4 h. Secreted IL-6 (left) and TNF-α (right) were measured by ELISA. Each bar is the mean of triplicates ± SD. Comparable results were obtained with cells from different BMMC cultures (*n* = 3 (**a**, **b**, **d**); *n* > 3 (**c**, **e**, **f**))

To learn about the functional interaction of Lyn and SHIP1 in such cells, we used Lyn-/- BMMCs (C57BL/6 x 129/Sv background), which showed severely decreased overall substrate Y-P (Fig. 1a), Ca^2+^ mobilization (Fig. 1b; Additional file 1: Figure S1), and attenuated degranulation in response to Ag stimulation (Fig. 1c) compared to wt cells. Next, we analyzed if Lyn was controlling SHIP1 Y-P and PKB phosphorylation in these cells as well. Indeed, SHIP1 Y-P was abrogated in Ag-triggered Lyn-/- BMMCs (Fig. 1d). In correlation, Ag-induced phosphorylation of PKB was augmented immensely in Lyn-/- BMMCs compared to wt cells (Fig. 1e). In line with increased PKB phosphorylation/activation, IL-6 and TNF-α production after Ag-mediated FcεRI crosslinking were strongly increased in Lyn-/- BMMCs compared to wt BMMCs (Fig. 1f). In conclusion, reduced Ca^2+^ mobilization and degranulation did manifest even in the presence of attenuated SHIP1 phosphorylation/activation in Lyn-deficient BMMCs.

### Normal differentiation of BMMCs double-deficient for Lyn and SHIP1

Suppressed Y-P of SHIP1 indicated reduced SHIP1 activation in Lyn-/- BMMCs, which would be expected to coincide with augmented PKB phosphorylation, Ca^2+^ mobilization, degranulation, and pro-inflammatory cytokine production [10, 12, 13]. However, only PKB phosphorylation and cytokine production were enhanced (Fig. 1). Thus, results shown in Fig. 1 suggested that Lyn deficiency concurs with reduced SHIP1 activity with respect to Ag-triggered PKB phosphorylation and cytokine production, but is dominant over the effect of suppressed SHIP1 Y-P/activation with respect to Ca^2+^ mobilization and degranulation. To follow this up, Lyn- and SHIP1-deficient mice were crossed and mice from the finally resulting F2 generation (wt, Lyn-/-, SHIP1-/-, and Lyn/SHIP1-double-deficient (dko) mice) were used for the differentiation of BMMCs. These cells differentiated comparably as shown by FACS analysis of 5-6 week old cells measuring expression of MC surface markers, FcεRI and Kit (Additional file 2: Figure S2A). Moreover, similar expression of the IL-33 receptor, ST2, and the myeloid cell marker, CD13, were observed (Additional file 2: Figure S2B). Deficiency of Lyn and SHIP1 was verified by Western blotting (Additional file 2: Figure S2C). Compared to wt cells, single- and double-deficient BMMCs proliferated significantly stronger, which is in agreement with a negative role of Lyn and SHIP1 in IL-3-induced proliferation of BMMCs and colony-forming progenitors (Additional file 2: Figure S2D; [15–17]). Moreover, analysis of degranulation in response to ionophore treatment showed no impairment in these cells (data not shown). In conclusion, Lyn and SHIP1 single- and double-deficiencies allowed BMMC differentiation and thus further analysis of these cells.

### Production of pro-inflammatory cytokines is controlled by both Lyn and SHIP1

**Fig. 2:**
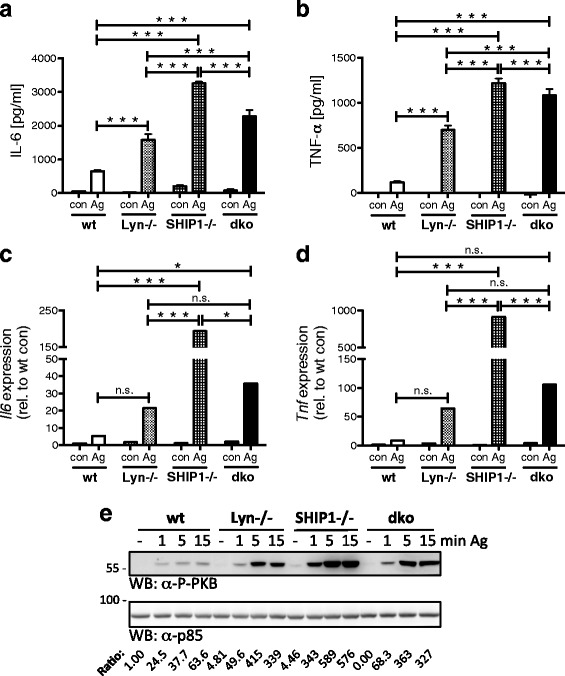
BMMCs deficient for Lyn and SHIP1 produce enhanced levels of pro-inflammatory cytokines in a PI3K-dependent manner. Wt, Lyn-/-, SHIP1-/-, and dko BMMCs were preloaded and starved overnight with 0.15 μg/ml IgE. **a, b** BMMCs were left unstimulated (con) or stimulated with Ag (20 ng/ml) for 4 h. Secreted IL-6 (**a**) and TNF-α (**b**) were measured by ELISA. Each bar is the mean of triplicates ± SD. For the response to further Ag concentrations see Additional file 3: Figure S3A & B. **c, d** BMMCs were left unstimulated (con) or stimulated with 20 ng/ml of Ag for 90 min. The amounts of *Il6* mRNA (**c**) and *Tnf* mRNA (**d**) were determined by RT-qPCR using the Pfaffl method. A comparison of analyses of different independent cell cultures is depicted in Additional file 4: Figure S4. For the response to further Ag concentrations see Additional file 3: Figure S3C & D. **e** BMMCs were left untreated (-) or stimulated with Ag (200 ng/ml) for the indicated time points. Whole-cell lysates were subjected to WB analysis with antibodies against P-PKB (upper panel) and p85 (loading control, lower panel). Densitometry was performed and relative expression levels are indicated under each band. Comparable results were obtained with cells from different BMMC cultures (*n* > 3 (**a**-**e**))

We have shown previously that SHIP1 restricts Ag-triggered production of IL-6 [13]. Moreover, Lyn deficiency resulted in increased IL-6/TNF-α production in response to Ag (Fig. 1; [6]). This suggested that Lyn/SHIP1 double-deficiency should also, or even more, cause augmented production of pro-inflammatory cytokines. To investigate this, wt, Lyn-/-, SHIP1-/-, and dko BMMCs were preloaded with DNP-specific IgE and subsequently stimulated with increasing concentrations of Ag (DNP-HSA). Production/secretion of IL-6 and TNF-α were determined by ELISA. Wt cells showed the typical bell-shaped dose-response pattern with strong suppression of IL-6/TNF-α production starting at 200 ng/ml Ag (Additional file 3: Figure S3A & B). Compared to wt cells, all single- and double-deficient BMMCs secreted markedly more IL-6/TNF-α at all Ag concentrations tested (Fig. 2a & b; Additional file 3: Figure S3A & B). A comparable response pattern was observed when Ag-induced production of *Il6* and *Tnf* mRNA was measured (Fig. 2c & d; Additional file 3: Figure S3C & D). Although pro-inflammatory cytokine mRNA was enhanced in Lyn-/- compared to wt BMMCs in response to optimal Ag concentration in several experiments (Additional file 4: Figure S4), statistical analysis did not yield significance (Fig. 2c & d). Augmented cytokine production correlated with enhanced PI3K-dependent phosphorylation of PKB in Lyn-/- and SHIP1-/- BMMCs (Fig. 1; [13]). Hence, we expected strong PKB phosphorylation in Ag-triggered dko BMMCs as well. Indeed, PKB phosphorylation in dko BMMCs was stronger than in wt and Lyn-/- cells, however, weaker than in SHIP1-/- BMMCs (Fig. 2e). Our results confirmed that Ag-triggered, PI3K-dependent *Il6*/IL-6 and *Tnf*/TNF-α production is controlled by Lyn and SHIP1. Moreover, Lyn co-deficiency seemed to weakly attenuate the SHIP1-/- phenotype, suggesting involvement of (a) stimulatory Lyn-dependent pathway(s). Furthermore, the quantitative differences between Lyn-/-, SHIP1-/-, and dko BMMCs indicated that single Lyn deficiency does not coincide with a complete blockade of SHIP1 function.

### Lyn deficiency dominates the SHIP1-deficient phenotype with respect to degranulation and preceding signaling events

**Fig. 3:**
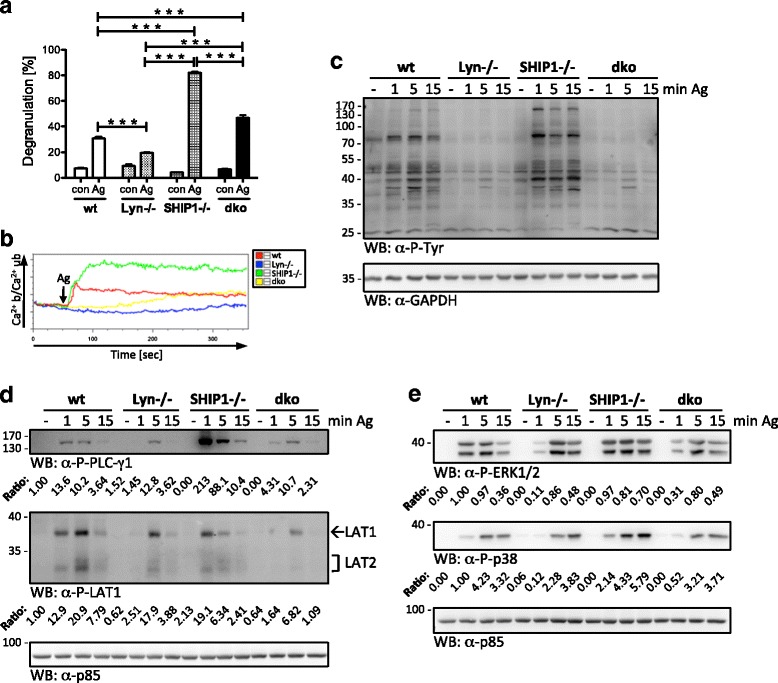
Lyn deficiency is dominant to combined SHIP1 deficiency concerning degranulation and relevant signaling events. Wt, Lyn-/-, SHIP1-/-, and dko BMMCs were preloaded and starved overnight with 0.15 μg/ml IgE. **a** Unstarved BMMCs were left untreated (con) or stimulated with Ag (20 ng/ml) for 20 min. Degranulation was determined by β-hexosaminidase assay. Each point is the mean of triplicates ± SD. For the response to different Ag concentrations see Additional file 5: Figure S5A. **b** Ca^2+^ mobilization was measured in wt, Lyn-/-, SHIP1-/-, and dko BMMCs for 6 min by flow cytometry as described in Fig. 1b. For the response to different Ag concentrations see Additional file 5: Figure S5B. **c** BMMCs were left untreated (-) or stimulated with Ag (20 ng/ml) for the indicated time points. Whole-cell lysates were subjected to WB analysis with anti-P-Tyr (upper panel) and anti-GAPDH (loading control, lower panel) antibodies. **d** BMMCs were left untreated (-) or stimulated with Ag (200 ng/ml) for the indicated time points. Whole-cell lysates were subjected to WB analysis with antibodies against P-PLC-γ1 (top panel), P-LAT1 (middle panel), and p85 (loading control, bottom panel). **e** BMMCs were treated as in (**d**). Whole-cell lysates were subjected to WB analysis with antibodies against P-ERK1/2 (top panel), P-p38 (middle panel), and p85 (loading control, bottom panel). Densitometry was performed and relative expression levels are indicated under each band. Comparable results were obtained with cells from different BMMC cultures (*n* = 3 (**b**, **d**); *n* = 2 (**c**); *n* > 3 (**a**, **e**))

In comparison to PKB phosphorylation and cytokine production, which were enhanced in both Lyn-/- and SHIP1-/- BMMCs (Fig. 1; [10]), degranulation and Ca^2+^ mobilization showed opposing trends (Fig. 1; [10]). We used wt, Lyn-/-, SHIP1-/-, and dko BMMCs to challenge the idea whether Lyn deficiency is dominant to a lack of SHIP1 expression with respect to degranulation and preceding signaling processes. IgE-preloaded cells were stimulated with increasing concentrations of Ag and, subsequently, release of the granular enzyme β-hexosaminidase was measured. In response to an optimal concentration of Ag (20 ng/ml DNP-HSA), SHIP1-/- BMMCs, as expected [10], degranulated far stronger than wt cells (Fig. 3a). In both comparisons (wt vs. Lyn-/- as well as SHIP1-/- vs. dko), Lyn-deficiency resulted in significantly reduced degranulation, particularly evident when comparing SHIP1-/- and dko BMMCs (Fig. 3a). In accordance with our initial analysis of wt and Lyn-/- BMMCs (Fig. 1), which showed reduced degranulation in Lyn-/- BMMCs in the absence of SHIP1 Y-P, co-deficiency of Lyn strongly suppressed degranulation of SHIP1-deficient BMMCs. This effect was especially obvious in response to high, supra-optimal Ag concentrations (Additional file 5: Figure S5A).

Store-operated Ca^2+^ entry is a prerequisite for Ag-triggered degranulation. Compared to wt BMMCs, Lyn-/- BMMCs have been reported to show a suppressed Ca^2+^ signal ([4–7, 9] and Fig. 1). SHIP1-/- BMMCs, on the other side, exerted markedly enhanced Ca^2+^ mobilization in response to Ag [10, 12]. This was corroborated in Ag (20 ng/ml DNP-HSA)-stimulated wt, Lyn-/-, and SHIP1-/- BMMCs (Fig. 3b). Interestingly, dko BMMCs showed no immediate Ca^2+^ flux (representing the time window most crucial for degranulation), as compared to wt and SHIP1-/- BMMCs. A slow and steady increase in intracellular Ca^2+^ content, however, was observed to reach the level seen in wt cells approximately 2 min after Ag addition (Fig. 3b). In principle, the same response pattern was found after stimulation with high to supra-optimal Ag concentrations (200 and 2000 ng/ml DNP-HSA), however, the delayed and sustained increase in intracellular Ca^2+^ was faster and reached higher levels (Additional file 5: Figure S5B). These data demonstrated that Lyn co-deficiency is able to dominate the SHIP1-deficient phenotype and that characteristics of the SHIP1-/- phenotype might prevail at higher Ag concentrations.

Next, we checked FcεRI-mediated signaling events preceding Ca^2+^ mobilization and degranulation. Wt, Lyn-/, SHIP1-/-, and dko BMMCs were stimulated with Ag for 1, 5, and 15 min and overall substrate Y-P was analyzed by anti-P-Tyr immunoblotting. Correlating with the qualitative response patterns for Ca^2+^ mobilization and degranulation, wt and SHIP1-/- BMMCs exerted strong substrate Y-P, whereas Y-P events were blunted in Lyn-/- and dko BMMCs (Fig. 3c). In order to understand how Lyn deficiency regulated degranulation as well as Ca^2+^ mobilization, we focused on the transmembrane adapter protein LAT1 as well as PLC-γ1. Both proteins are involved in the induction of Ca^2+^ mobilization [18] and the accepted model of FcεRI signaling involves Lyn in the phosphorylation of both in an indirect manner: LAT1 via Lyn-mediated Syk activation and PLC-γ1 via Lyn-mediated Btk activation [3]. Most notable with respect to the induction of Ca^2+^ release was the 1 min time point. Whereas LAT1 phosphorylation was induced in wt and SHIP1-/- BMMCs 1 min after Ag addition, this response was only marginal in Lyn-/- and dko BMMCs (Fig. 3d), fitting to the observed Ca^2+^ signals (Fig. 3b). Whereas in wt cells P-LAT1 was strongest at 5 min and still visible after 15 min, SHIP1-/- BMMCs showed highest LAT1 Y-P at 1 min and considerably reduced signals at 5 and 15 min. Corresponding differences were found between Lyn-/- and dko BMMCs with dko cells showing weaker P-LAT1 signals than Lyn-/- BMMCs particularly at 5 and 15 min (Fig. 3d). The phospho-specific anti-LAT1 antibody used in this analysis is known to also recognize phosphorylated LAT2 (a.k.a. NTAL) [19]. P-LAT2 followed the pattern described for P-LAT1 (Fig. 3d), suggesting comparable dependence on presence of Lyn and/or SHIP1. With respect to signal initiation, PLC-γ1 Y-P showed a comparable pattern to LAT1 Y-P. Ag-triggered wt and SHIP1-/- BMMCs showed PLC-γ1 Y-P within 1 min, whereas in Lyn-/- and dko BMMCs only marginal signals were detectable (Fig. 3d). Conspicuously, PLC-γ1 Y-P was by far strongest in SHIP1-/- BMMCs, suggesting that PIP_3_-dependent signaling processes promote PLC-γ1 Y-P, at least in SHIP1-deficient cells [20]. The 160 kDa protein, heavily tyrosine-phosphorylated in SHIP1-/- BMMCs (Fig. 3c), most likely represented PLC-γ1. In conclusion, Lyn co-deficiency dominated the SHIP1-/- phenotype of BMMCs concerning degranulation and preceding signaling events.

In response to FcεRI engagement, LAT1 scaffolds several protein complexes at the plasma membrane, amongst them Grb2/Sos resulting in phosphorylation/activation of the MAPKs ERK1/2. Indeed, early phosphorylation (1 min) of ERK1/2 was reduced in Lyn-/- and dko BMMCs compared to wt and SHIP1-/- cells, respectively, correlating with Y-P of LAT1. A comparable pattern was observed for Ag-triggered phosphorylation of the MAPK p38 (Fig. 3e). Attenuated early, Ag-triggered phosphorylation of ERK1/2 and p38 in Lyn-/- BMMCs was in agreement with previous work by Xiao et al. [4].

### Lyn deficiency reveals Ca^2+^-dependence of Ag-triggered activation of the NFκB pathway

**Fig. 4:**
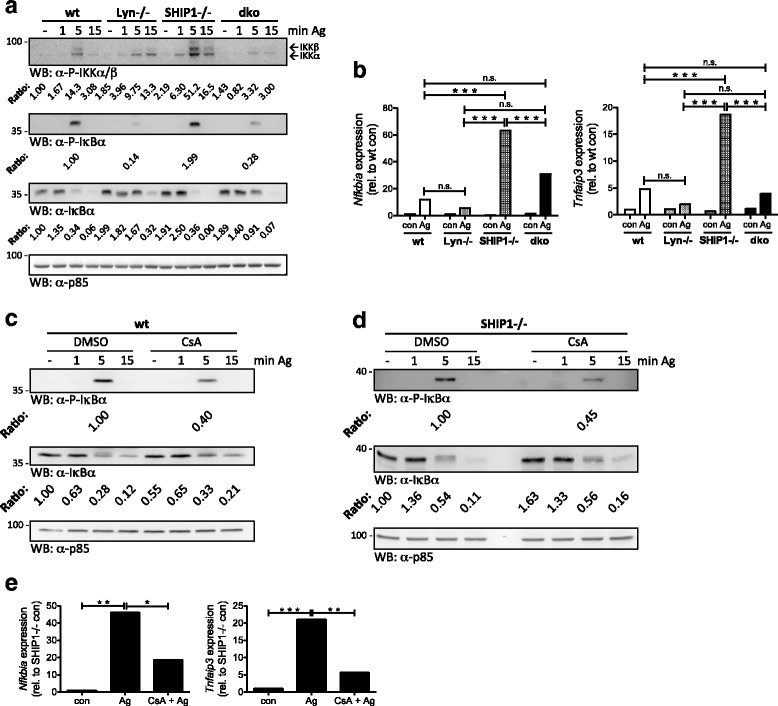
Lyn/Ca^2+^/calcineurin-dependent activation of the NFκB pathway in Ag-triggered BMMCs. Wt, Lyn-/-, SHIP1-/-, and dko BMMCs were preloaded and starved overnight with 0.15 μg/ml IgE. **a** BMMCs were left untreated (-) or stimulated with Ag (200 ng/ml) for the indicated time points. Whole-cell lysates were subjected to WB analysis with antibodies against P-IKKα/β (top panel), P-IκBα (second panel from top), IκBα (third panel from top), and p85 (loading control, bottom panel). Densitometry was performed and relative expression levels are indicated under each band. **b** BMMCs were left unstimulated (con) or stimulated with Ag (20 ng/ml) for 90 min. The amounts of *Nfkbia* mRNA (left panel) and *Tnfaip3* mRNA (right panel) were measured by RT-qPCR. A comparison of analyses of different independent cell cultures is depicted in Additional file 6: Figure S7. For the response to different Ag concentrations see Additional file 7: Figure S6A. **c** Wt BMMCs, pretreated with vehicle (DMSO) or 100 nM CsA for 30 min, were left unstimulated (-) or stimulated with Ag (20 ng/ml) for the indicated time points. Whole-cell lysates were subjected to WB analysis with antibodies against P-IκBα (top panel), IκBα (middle panel), and p85 (loading control, bottom panel). **d** IgE-loaded SHIP1-/- BMMCs, pretreated with vehicle (DMSO) or 100 nM CsA for 30 min, were left unstimulated (-) or stimulated with Ag (20 ng/ml) for the indicated time points. Whole-cell lysates were subjected to WB analysis with antibodies against P-IκBα (top panel), IκBα (middle panel), and p85 (loading control, bottom panel). Densitometry was performed and relative expression levels are indicated under each band. A statistical analysis of the CsA effects is shown in Additional file 7: Figure S6C & D. **e** SHIP1-/- BMMCs, pretreated with vehicle (DMSO) or 100 nM CsA for 30 min, were left unstimulated (con) or stimulated with Ag (20 ng/ml) for 90 min. The amounts of *Nfkbia* mRNA (left panel) and *Tnfaip3* mRNA (right panel) were measured by RT-qPCR. Comparable results were obtained with cells from different BMMC cultures (*n* = 3 (**a**, **b**); *n* = 3 (**c**, **d**); *n* > 3 (**e**))

NFκB activation downstream of the FcεRI depends on the adaptor protein Bcl-10 and the paracaspase MALT1. It is crucial for Ag-stimulated MC activation, particularly with respect to pro-inflammatory (*Il6* and *Tnf*) gene transcription [21]. Here, we analyzed the impact of Lyn deficiency on NFκB activation in the context of wt and SHIP1-/- BMMCs. Wt, Lyn-/-, SHIP1-/-, and dko BMMCs were stimulated with Ag and phosphorylation of IKKα/β was analyzed by immunoblotting. Particularly evident was the reduced phosphorylation of IKKβ after 5 min of Ag stimulation in Lyn-/- and dko BMMCs (Fig. 4a). Additionally, phosphorylation as well as subsequent degradation of IκBα were studied. Comparable phosphorylation of IκBα was observed after 5 min in wt and SHIP1-/- BMMCs correlating with degradation/disappearance of IκBα (5 and 15 min) (Fig. 4a). In accordance with previous data, basal IκBα expression in SHIP1-/- BMMCs was stronger than in wt cells [13], and a comparable behavior was observed in dko and Lyn-/- BMMCs. The underlying mechanism, however, is unknown at present. Unexpectedly, IκBα phosphorylation was markedly reduced and degradation retarded in Lyn-/- and dko compared to wt and SHIP1-/- BMMCs, respectively (Fig. 4a), despite augmented production of IL-6 and TNF-α (Fig. 2a & b). Thus, Lyn plays a positive regulatory role for NFκB activation in Ag-triggered wt and SHIP1-/- BMMCs.

Due to the discrepancy between IκBα phosphorylation/degradation and pro-inflammatory cytokine production, we next determined if the observed pattern of IκBα phosphorylation/degradation proceeded to transcription of typical NFκB target genes. The genes for IκBα (*Nfkbia*) and the deubiquitinating enzyme A20 (*Tnfaip3*) have been demonstrated to be transcribed in an NFκB-dependent manner [22, 23]. Therefore, production of *Nfkbia* and *Tnfaip3* mRNA in Ag-stimulated (90 min) wt, Lyn-/-, SHIP1-/-, and dko BMMCs were measured using RT-qPCR. Indeed, differences in respective mRNA production in response to an optimal Ag concentration (20 ng/ml) followed the pattern observed for IκBα phosphorylation and degradation (Fig. 4b). Lyn-/- and dko BMMCs produced markedly less *Nfkbia* and *Tnfaip3* mRNA compared to wt and SHIP1-/- BMMCs, respectively, with the most dramatic difference seen between SHIP1-/- and dko cells (Fig. 4b). Though *Nfkbia* and *Tnfaip3* mRNA production was increased in wt vs. Lyn-/- BMMCs in response to 20 ng/ml Ag in several experiments (Additional file 6: Figure S7), combined differences did not yield statistical significance (Fig. 4b). In SHIP1-/- BMMCs, particularly high levels of *Nfkbia* and *Tnfaip3* mRNAs were measured, in line with the strong negative regulation of the NFκB pathway by SHIP1 [13]. In agreement with a negative regulatory role of SHIP1 particularly in response to high doses of Ag [10, 24], dko BMMCs produced higher levels of *Nfkbia* and *Tnfaip3* mRNA compared to wt cells (Additional file 7: Figure S6A).

Interestingly, the observed pattern of NFκB activation strongly correlated with Ag-induced Ca^2+^ mobilization and LAT1/PLC-γ1 Y-P in the cell types under investigation (compare to Fig. 3). Therefore, we speculated that Ag-triggered NFκB activation contains a Ca^2+^-dependent signaling step. Consequently, wt and SHIP1-/- BMMCs were left untreated or stimulated with Ag for 5 min after short-term EDTA- or vehicle treatment and phosphorylation/degradation of IκBα was analyzed. In both cell types, EDTA-mediated depletion of extracellular Ca^2+^ (and thus abrogation of store-operated Ca^2+^ entry) resulted in attenuated IκBα phosphorylation/degradation compared to control treatment (Additional file 7: Figure S6B). This pointed to a positive function of store-operated Ca^2+^ entry upstream of FcεRI-mediated IκBα phosphorylation. In this respect, the Ca^2+^/calmodulin-dependent phosphatase calcineurin has been demonstrated in T helper cells to contribute to activation of the NFκB pathway [25]. Our further analysis revealed that Ag-triggered IκBα phosphorylation/degradation was significantly attenuated in wt and SHIP1-/- BMMCs pre-incubated with the calcineurin inhibitor cyclosporine A (CsA) (Fig. 4c & d; Additional file 7: Figure S6C & D). Consequently, Ag-induced production of *Nfkbia* and *Tnfaip3* mRNA were significantly suppressed in CsA-pretreated SHIP1-/-BMMCs (Fig. 4e). In conclusion, by governing FcεRI-mediated Ca^2+^ mobilization, Lyn acts as positive regulator of calcineurin-controlled NFκB activation and transcription of NFκB-dependent genes.

### Identification of genes transcribed in a Lyn/Ca^2+^/calcineurin-dependent manner

**Fig. 5:**
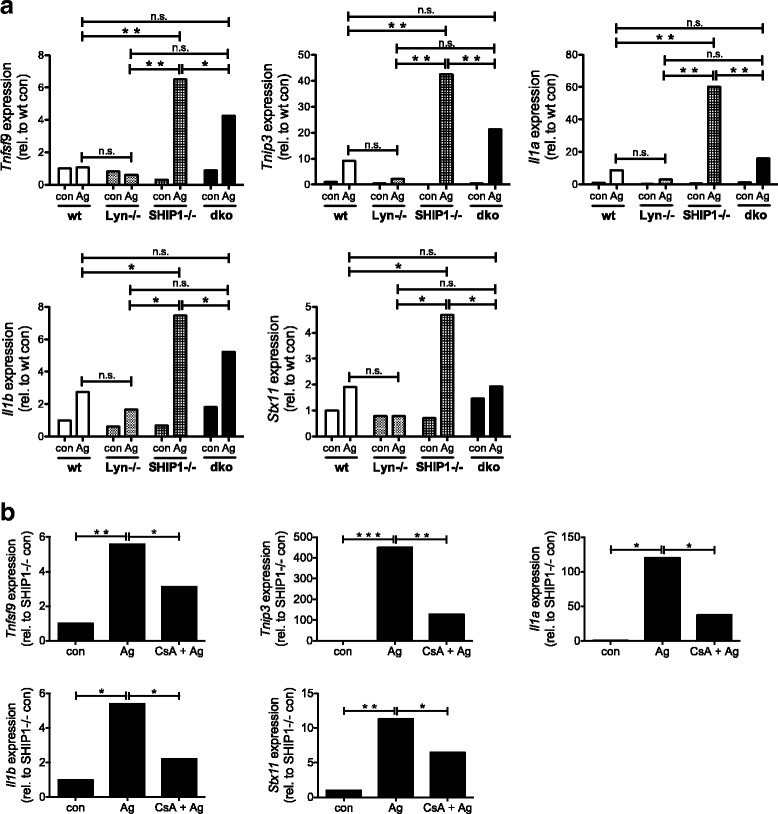
Lyn/Ca^2+^/calcineurin-dependent signaling controls Ag-induced transcription of various inflammatory genes. BMMCs were preloaded and starved overnight with 0.15 μg/ml IgE. **a** Wt, Lyn-/-, SHIP1-/-, and dko BMMCs were left untreated (con) or stimulated with 20 ng/ml of Ag for 90 min. The amounts of *Tnfsf9* mRNA, *Tnip3* mRNA, *Il1a* mRNA, *Il1b* mRNA, and *Stx11* mRNA were measured by RT-qPCR. A comparison of analyses of different independent cell cultures is depicted in Additional file 8: Figure S8. **b** SHIP1-/- BMMCs, pretreated with vehicle or 100 nM CsA for 30 min, were left unstimulated (con) or stimulated with 200 ng/ml Ag for 90 min. The amounts of *Tnfsf9* mRNA, *Tnip3* mRNA, *Il1a* mRNA, *Il1b* mRNA, and *Stx11* mRNA were measured by RT-qPCR. Comparable results were obtained with cells from different BMMC cultures (*n* = 3 (**a**); *n* > 3 (**b**)

To learn more about Lyn/Ca^2+^/calcineurin-dependent gene transcription, a preliminary Affymetrix transcriptome analysis was carried out comparing Ag-triggered SHIP1-/- and dko BMMCs. Transcription of *Tnip3*, *Il1a*, *Il1b*, *Tnfsf9*, and *Stx11* appeared to be significantly reduced in dko vs. SHIP1-/- BMMCs (data not shown). For verification, wt, Lyn-/-, SHIP1-/-, and dko BMMCs were stimulated with Ag for 90 min and mRNA production was analyzed by RT-qPCR. Indeed, in response to 20 ng/ml Ag, wt and SHIP1-/- BMMCs showed stronger production of *Tnip3*, *Il1a*, *Il1b, Tnfsf9*, and *Stx11* mRNAs compared to Lyn-/- and dko BMMCs, respectively, though statistical significance was only reached in SHIP1-/- cells (Fig. 5a; Additional file 8: Figure S8). Again, SHIP1-/- BMMCs produced particularly high levels of the respective mRNAs, which is in line with the dominant control of the NFκB pathway by SHIP1 [13]. In agreement with the calcineurin-mediated regulation of *Nfkbia* and *Tnfaip3* mRNA production described above (Fig. 4e), CsA treatment significantly attenuated transcription of *Tnip3*, *Il1a*, *Il1b*, *Tnfsf9*, and *Stx11* in SHIP1-/- BMMCs (Fig. 5b). Interestingly, in addition to *Nfkbia* and *Tnfaip3*, *Tnip3*, *Il1a*, and *Il1b* are also known to be transcribed in an NFκB-dependent fashion [26–28]. These data indicate that a Lyn/Ca^2+^/calcineurin/NFκB-dependent signaling pathway is functional for the regulation of Ag-triggered transcription of various genes that are most likely involved in pro-inflammatory processes.

## Discussion

In response to FcεRI crosslinking, both Lyn-/- and SHIP1-/- BMMCs show stronger production of pro-inflammatory cytokines and enhanced phosphorylation of PKB compared to wt BMMCs [6, 13]. These parallel phenotypes conformed to the finding that Lyn is able to phosphorylate SHIP1, thereby contributing to SHIP1 activation [5]. This functional Lyn/SHIP1 interaction constitutes a feedback inhibition of PI3K/PIP_3_-mediated signals, in particular PKB-driven cytokine production. However, whereas SHIP1-/- BMMCs degranulated markedly stronger than wt cells, Lyn-/- BMMCs appeared to exert varying degranulation phenotypes (as detailed in the Introduction section). In several reports as well as in this study, Lyn-/- BMMCs were described, which showed enhanced PKB phosphorylation and cytokine production in response to Ag, however, degranulated less compared to wt cells [6, 9, 14]. Since reduced degranulation did not concur with a SHIP1-deficient phenotype, the question arose if the functional Lyn/SHIP1 interaction might be absent in the respective wt BMMCs and Lyn might not be involved in these cells in the phosphorylation/activation of SHIP1. However, our analysis revealed that in these cells Lyn was still responsible for Ag-triggered Y-P of SHIP1 and the Lyn-/- BMMCs showed abrogated Y-P of SHIP1.

The combination of reduced degranulation and suppressed SHIP1 Y-P was rather unexpected. We set out to analyze this phenomenon more closely and hence generated wt, Lyn-/-, SHIP1-/-, and dko BMMCs. Based on our initial analysis of wt and Lyn-/- BMMCs as well as our experience with SHIP1-/- BMMCs, we expected that dko cells would also show enhanced PKB phosphorylation and pro-inflammatory cytokine production (IL-6 and TNF-α). Indeed, these PI3K-dependent responses were markedly increased in Lyn-/- and SHIP1-/- as well as in dko BMMCs compared to wt cells.

With respect to degranulation, the outcome of the experiment was less obvious, since dominance of both the Lyn-/- as well as the SHIP1-/- phenotype was conceivable. The result, however, was very clear-cut. In dko cells, the Lyn-/- phenotype dominated the SHIP1-/- phenotype with respect to Ag-triggered Y-P of LAT1/2 and PLC-γ1, Ca^2+^ mobilization, and degranulation. Both PLC-γ-regulated signaling events, Ca^2+^ mobilization and diacylglycerol (DAG)-dependent PKC activation, are important for degranulation [29]. Therefore, our data suggest that the extent of limitation in PLC-γ activation might be overcompensating the strong increase in PI3K pathway activity in both Lyn-/- and dko BMMCs. Interestingly, dko BMMCs degranulated significantly stronger than Lyn-/- cells, though showing reduced LAT Y-P. One might suggest that phosphorylation-dependent recruitment of a negative regulator other than SHIP1 might be hampered in dko compared to Lyn-/- cells and thus contribute to augmented degranulation. In this respect, Salek et al. demonstrated that LAT can be implicated in the organization of negative feedback signals [30]. Moreover, the SFK Fyn has been shown to be a positive regulator of Ag-triggered degranulation [8] and to exert stronger expression and/or higher activity in certain studies of Lyn-/- BMMCs [5, 31]. However, analysis of STAT5 Y-P in response to Ag, which has been demonstrated by Ryan and coworkers to be strictly dependent on Fyn [32], revealed attenuated responses in Lyn-/- and dko BMMCs compared to wt cells (data not shown). Furthermore, expression levels of Fyn in these cell types were comparable (data not shown). Hence, reduced Fyn activity in Lyn-/- and dko BMMCs could contribute to the observed degranulation phenotype.

Additionally, one cannot exclude the involvement of another pathway that is activated in a Lyn/LAT-dependent, but PLC-γ-independent manner and is positively impacting on degranulation. In this respect, it is important to note that i) ERK1/2 phosphorylation is reduced at the 1 min time point in Lyn-/- and dko cells compared to wt and SHIP1-/- BMMCs, respectively; ii) degranulation is an immediate response with early signals (1 min) being crucial for the whole process [10]; and iii) ERK1/2 activation has been shown to be involved in the positive control of degranulation [33, 34]. Thus, increased activation of the PI3K pathway in Lyn-/- and dko BMMCs might not be able to compensate for a combination of suppressed Ca^2+^ mobilization/DAG generation and reduced MAPK activation at the 1 min time point.

Interestingly, ERK1/2 activation in Lyn-/- and dko BMMCs was only suppressed at the 1 min time point, suggesting that ERK1/2 phosphorylation passes through two phases, an immediate LAT1-dependent phase followed by a sustained LAT1-independent phase. In this respect, LAT-independent activation of ERK1/2 in T-cells via a Bam32/PLC-γ1/Pak1 complex has been reported [35]. This represents a Rac-independent mechanism for Pak1 activation, which has been shown to employ a scaffolding function of PLC-γ1. Concurrently, we have demonstrated in Ag-stimulated BMMCs that Pak1 phosphorylation was only partly inhibited by the use of Rac-specific lethal toxin of *Clostridium sordellii* indicating a second Rac-independent pathway for Pak1 activation in BMMCs as well [36].

Another unexpected outcome of our study was the Lyn/Ca^2+^/calcineurin-dependent activation of the NFκB pathway, as measured by IKKα/β phosphorylation, IκBα phosphorylation/degradation, and activation of well-known NFκB target genes (e.g. *Nfkbia* and *Tnfaip3*). Interestingly, Baba et al. found in Stim1-/- fetal liver-derived MCs, which showed severely reduced Ag-triggered Ca^2+^ mobilization and TNF-α production, that IκBα phosphorylation and degradation as well as p65 nuclear translocation were reduced [37]. Calcineurin is well known for dephosphorylating the transcription factor NFAT in a Ca^2+^/calmodulin-dependent manner, which subsequently enters the nucleus and regulates transcription of its target genes (e.g. *Il2* in T-lymphocytes or *Tnf* in MCs [38]). However, non-NFAT targets have been recently reported for calcineurin [25, 39]. The pharmacological inhibitor CsA does not allow for a differentiation between various calcineurin substrates. However, another NFAT activation-blocking inhibitor, INCA-6, does not inhibit the catalytic activity of calcineurin, but instead blocks the interaction between calcineurin and NFAT [40]. In agreement with its dependence on NFAT and NFκB, Ag-triggered TNF-α production in wt BMMCs was stronger suppressed by an optimal concentration of CsA than by an optimal dose of INCA-6 (Additional file 9: Figure S9A). In contrast, induction of *Nfkbia* after 30 min of Ag stimulation was only blocked by CsA, indicating that calcineurin regulates early NFκB activation via an NFAT-independent mechanism in wt BMMCs (Additional file 9: Figure S9B). Bcl-10, in a complex with MALT1, has been demonstrated to be involved in NFκB activation in BMMCs in response to Ag stimulation [21]. Moreover, calcineurin has been shown in T helper cells to control IκBα phosphorylation by the IKK complex via dephosphorylation of Bcl-10 [25]. Hence, CsA-treatment of stimulated T helper cells resulted in increased Bcl-10 phosphorylation at Ser138. Though it was tempting to presume an analogous mechanism in BMMCs, we did not observe an increase of Bcl-10 Ser138 phosphorylation in Ag-triggered BMMCs pretreated with CsA (data not shown). Thus, so far we are not able to specify the calcineurin target responsible for the observed effects. Nevertheless, our pharmacological approach (CsA vs. INCA-6) indicates that the effect is largely independent of NFAT.

Still there is no satisfying explanation why different Lyn-/- BMMCs show opposing effects in degranulation when compared to wt cells. Yamashita et al. offered differences in genetic background [6], however, as detailed in the Introduction section, there are also reports which do not support this notion and thus, it is rather unlikely that different genetic backgrounds are the only reason for this discrepancy. Generation of BMMCs involves a 4-6 weeks-differentiation/cultivation period in medium containing, amongst others, FCS and cytokines (mostly IL-3). FCS, containing a plethora of proteins and low-molecular weight substances, which can be quantitatively and/or qualitatively different between manufacturers and distinct lot numbers, very likely can contribute to phenotypic differences. Though all cells studied by different groups, without doubt, are wt and Lyn-/- BMMCs, they most likely contain somewhat different proteomes, causing quantitative and/or qualitative differences in activation phenotypes. Thus, the more important question might be, why is the Lyn-/- phenotype so susceptible for changes in differentiation conditions? As already mentioned, Lyn has dichotomous functions in Ag-stimulated MCs, positive ones (e.g. phosphorylation of the FcεRI ITAMs) and negative ones (e.g. phosphorylation of SHIP1). Since all these regulatory mechanisms depend on the correct interplay of proteins and assembly of protein complexes, minute proteomic changes might lead to differences in phenotypes. In this line, BMMCs express various SFKs in addition to Lyn (e.g. Fyn, Src, and Hck), which are known to exert opposing and/or redundant functions [8, 41]. Depending on their level of expression, compensatory actions in Lyn-deficient BMMCs might be possible.

A further topic worth to be discussed is the fact that both Lyn-/- and dko BMMCs are able to degranulate (albeit significantly weaker than wt and SHIP1-/- BMMCs, respectively), despite their apparent lack of Ca^2+^ mobilization within the immediate phase (1 min) after FcεRI activation. Using pharmacological and genetic tools, the importance of Ca^2+^ influx in MC activation and degranulation has been demonstrated [29, 37, 42]. Whereas Ca^2+^ mobilization appears necessary for granule-plasma membrane fusion, a further Lyn- and Ca^2+^-independent process, namely microtubule formation, has been shown to drive granule translocation to the plasma membrane [42]. The concentration of cytosolic Ca^2+^ necessary for degranulation of BMMCs has not been defined yet. However, our data suggest that a minimal Ca^2+^ influx is sufficient given that additional mechanisms, such as PI3K pathway, are adequately activated (see dko cells). This also impacts on how we look at enhanced degranulation in SHIP1-/- BMMCs. So far, augmented Ag- or SCF-triggered degranulation of SHIP1-/- BMMCs was thought to depend, at least in part, on the dramatically increased Ca^2+^ mobilization observed in these cells [12, 43]. However, both enhanced calcium mobilization and augmented degranulation most likely are just two parallel phenotypes of SHIP1-/- BMMCs without a principle linear relationship. This was best documented by the comparison between wt and dko BMMCs. The latter degranulated significantly stronger than wt cells though Ca^2+^ mobilization in the immediate phase was only marginal.

## Conclusions

Different laboratories have reported on Lyn-/- BMMCs developing opposing degranulation phenotypes and this is, most likely, due to varying differentiation and culture conditions. In summary, we provide evidence that even in Lyn-/- BMMCs showing reduced degranulation, the Y-P of SHIP1 was abrogated. Concordantly, in Lyn/SHIP1 double-deficient BMMCs the Lyn-/- phenotype was dominant over the SHIP1-/- phenotype with respect to Ag-triggered degranulation and preceding signaling pathways. Additionally, using these cells a novel Lyn/Ca^2+^/calcineurin-dependent pathway controlling expression of NFκB-regulated pro-inflammatory genes was revealed.

## Methods

### Animals and cell culture

The following mutant mice were used: Lyn-/- mice (*Lyn*^*-/-*^) [44] and SHIP1-/- mice (*Inpp5d*^*-/-*^) [17]. Mating of these mice resulted in *Lyn*^*+/-*^*Inpp5d*^*+/-*^ F1 progeny. F1 mice were mated to obtain wt, Lyn-/-, SHIP1-/-, and Lyn-/-SHIP1-/- mice *(*referred to as dko). All mice were on a mixed C57BL/6 x 129/Sv background and littermates were used. Experiments were performed in accordance with German legislation governing animal studies and following the Principles of Laboratory Animal Care. Mice are held in the Institute of Laboratory Animal Science, Medical Faculty of RWTH Aachen University. The Institute holds a licence for husbandry and breeding of laboratory animals from the Veterinary Office of the Städteregion Aachen (Administrative District). The Institute follows a Quality Management System, which is certified according to DIN ISO 9001/2008. Every step in this project involving mice was reviewed by the animal welfare officer.

For the generation of BMMCs, BM cells (1x10^6^/ml) from 6- to 8-week-old mice were cultured (37 °C, 5 % CO_2_) as single cell suspensions in RPMI 1640 medium containing 15 % FCS, 1 % X63Ag8-653-conditioned medium (source of IL-3 [45]), 2 mM L-glutamine, 1x10^−5^ M 2-mercaptoethanol, 50 units/ml penicillin, and 50 mg/ml streptomycin. Every week, nonadherent cells were reseeded at 5x10^5^ cells/ml in fresh medium. After 4–6 weeks in culture, more than 95 % of the cells were Kit- and FcεRI-positive as assessed by FACS using phycoerythrin-labeled anti-mouse Kit antibody (clone 2B8; BD Biosciences, Heidelberg, Germany) and FITC-labeled rat anti-mouse IgE antibody (Southern Biotechnology Associates, Birmingham, AL, USA), respectively. Expression on the MC surface of ST2 was detected by anti-mouse ST2-FITC, clone DJ8 (MD Biosciences, Egg/Zürich, Switzerland) and expression of surface CD13 by anti-mouse CD13-PE, clone R3-242 (BD Biosciences, Heidelberg, Germany) using flow cytometry.

### Antibodies and reagents

Antibodies directed against IκBα, phospho-ERK1/2 (Thr202/Tyr204), phospho-IκBα (Ser32), phospho-IKKα/β (Ser176/180), phospho-LAT1 (Tyr191), phospho-p38 (Thr180/Tyr182), phospho-PKB (Ser473), phospho-tyrosine (P-Tyr-100) were from Cell Signaling Technology (Frankfurt, Germany). Antibodies against phospho-PLC-γ1 (Tyr783; sc-12943-R), GAPDH (6C5) and SHIP1 (P1C1) were obtained from Santa Cruz (Heidelberg, Germany) and antibodies against PI3K p85 (06-195) from EMD Millipore (Merck, Darmstadt, Germany). DNP-HSA containing 30-40 moles DNP per mole albumin and monoclonal IgE with specificity for DNP (SPE-7) were purchased from SIGMA (Deisenhofen, Germany). SPE-7 is a potent cytokinergic IgE and free SPE-7 was shown to cross-link FcεRI-bound SPE-7 by Fv-Fv interactions [46]. For this cytokinergic action the used concentration of SPE-7 (>1.0 μg/ml) had to be high enough to saturate all FcεRIs [46]. Therefore, to prevent interference with such cytokinergic action, we used only 0.15 μg/ml SPE-7 for preloading overnight for all experiments. 0.15 μg/ml SPE-7 was shown to be not sufficient to saturate all FcεRIs on BMMCs (data not shown). DMSO was obtained from Carl Roth GmbH & Co (Karlsruhe, Germany), Wortmannin from Calbiochem (Schwalbach, Germany), Cyclosporine A from Enzo Lifesciences (Lörrach, Germany), and INCA-6 from Tocris Bioscience (Wiesbaden-Nordenstadt, Germany).

### Degranulation assay

Cells were preloaded with 0.15 μg/ml IgE overnight. Subsequently, cells were washed and resuspended in Tyrode’s buffer (130 mM NaCl, 5 mM KCl, 1.4 mM CaCl_2_, 1 mM MgCl_2_, 5.6 mM glucose, and 0.1 % bovine serum albumin in 10 mM Hepes, pH 7.4). Cells were then adapted to 37 °C for 20 min and treated for 20 min at 37 °C as indicated. The percentage of degranulation was determined by measuring the release of β-hexosaminidase [7].

### Calcium measurement

IgE-preloaded BMMCs were resuspended at 10^7^ cells/ml in RPMI 1640 containing 1 % FCS, 1.3 μM Fluo-3 AM, 2.7 μM Fura Red AM, and 0.1 % pluronic F-127 (Life Technologies, Darmstadt, Germany) and incubated for 45 min at 37 °C. Cells were then resuspended in RPMI 1640 containing 1 % FCS, stimulated as indicated, and analyzed in a FACSCalibur flow cytometer (BD Biosciences, Heidelberg, Germany). Subsequently, FACS profiles were converted to line graph data using the FlowJo analysis software (Treestar, Ashland, OR, USA).

### Cytokine ELISAs

Mouse IL-6 ELISAs (BD Pharmingen, Heidelberg, Germany) and mouse TNF-α ELISAs (R&D Systems, Wiesbaden-Nordenstadt, Germany) were carried out according to the manufacturer’s instructions. Concentrations of cytokines varied between experiments/cell cultures. Qualitative differences or similarities, however, were consistent throughout the study.

### Immunoprecipitation and immunoblotting

Cells were preloaded with DNP-specific IgE overnight, washed with PBS, and resuspended in RPMI/0.1 % bovine serum albumin. Cells were adapted to 37 °C and stimulated for the indicated times with Ag (DNP-HSA). Cells were then pelleted and solubilized with 0.5 % IGEPAL and 0.5 % deoxycholate in phosphorylation solubilization buffer at 4 °C [47]. After normalizing for protein content, whole-cell lysates were subjected to SDS-PAGE or to immunoprecipitation and subsequent Western blot (WB) analysis. For immunoprecipitation, whole-cell lysates were incubated overnight at 4 °C using the indicated antibody, followed by incubation with 50 μl protein G-Sepharose beads (GE Healthcare, Freiburg, Germany) for 2 hours at 4 °C. The thoroughly washed precipitate was separated by SDS-PAGE and analyzed by immunoblotting.

### RT-qPCR

Total RNA from 4x10^6^ cells was extracted using RNeasy Mini Kit (Qiagen) according to the manufacturer’s instructions. RNA (1 μg) was subjected to reverse transcription using Random hexamers (Roche) and Omniscript Kit (Qiagen) according to the manufacturer’s instructions. Quantitative PCR was performed with 10 pmol of specific primers on a Rotorgene (Corbett Life Science) by using the Sybr green reaction mix (Bioline #QT650-02). Expression was normalized to the reference gene *Gusb* (Qiagen). Relative expression ratios including primer efficiencies were calculated according to Pfaffl [48]. Values are depicted relative to the values obtained in DMSO-pretreated wt cells (when all four genotypes were analyzed).

Primers: *Tnfaip3* fwd AAA CCA ATG GTG ATG GAA ACT G, rev GTT GTC CCA TTC GTC ATT CC, eff.: 2.04011; *Il6* fwd TCC AGT TGC CTT CTT GGG AC, rev GTG TAA TTA AGC CTC CGA CTT G, eff.: 2.00934; *Il1a* fwd ACG TCA AGC AAC GGG AAG AT, rev ACG TCA AGC AAC GGG AAG AT, eff.: 1.88729; *Il1b* fwd AAC CTG CTG GTG TGT GAC GTT C, rev CAG CAC GAG GCT TTT TTG TTG T, eff.: 1.99029; *Nfkbia* fwd CTC CCC CTA CCA GCT TAC CT, rev TAG GGC AGC TCA TCC TCT GT, eff.: 1.96052; *Stx11* fwd GCA GGG CAA GTG GGA TGT AT, rev CTA GCA CGG CCA TCT GTA GG, eff.: 2.22455; *Tnf* fwd AGC ACA GAA AGC ATG ATC CGC, rev TGC CAC AAG CAG GAA TGA GAA G, eff.: 2.18540; *Tnfsf9* fwd CTC CTG TGT TCG CCA AGC TA, rev CGG GAC TGT CTA CCA CCA AC, eff.: 1.83171; *Tnip3* fwd AGG ATG CCT TGA CCA TTG GG, rev GTG CTG TAC ACG TGG AGG AA, eff.: 2.16539; *Gusb* (Qiagen) cat. # QT00176715; eff.: 2.01478.

### Statistical analysis

The statistical analysis of the data was performed with the program JMP version 10.1 (SAS, Cary NC, USA). The data was obtained from n independent experiments, being n indicated in the respective figure legends. The data was then analyzed via a linear mixed model (LMM) using the approach of the least squares (LS) mean differences. P values were calculated by an unpaired two-tailed Student’s t test. These calculated p values were considered statistically significant according to the following:

* < 0.05, ** < 0.005, and *** < 0.0005; n.s. stands for not significant.

## Endnotes

The official gene name for murine SHIP1 is *Inpp5d*. For a better understanding, we used the term SHIP1-/- for SHIP1-deficient cells throughout the manuscript and did not use the notation *Inpp5d*^*-/-*^ cells.

## Additional files

Additional file 1: Figure S1. Impaired Ca^2+^ mobilization in Lyn-/- BMMCs is independent of the stimulus concentration. Wt and Lyn-/- BMMCs were preloaded and starved overnight with 0.15 μg/ml IgE. Ca^2+^ mobilization was measured for 4 min by flow cytometry using the Ca^2+^-sensitive fluorescent dyes fluo-3 (Ca^2+^ b) and fura red (Ca^2+^ ub). Steady-state fluorescence was assessed for 1 min before BMMCs were stimulated with the indicated Ag concentrations. The arrow marks the time point of stimulus addition. Comparable results were obtained with cells from different BMMC cultures (*n* = 3). (PDF 289 kb) Additional file 2: Figure S2. Wt, Lyn-/-, SHIP1-/- and dko BMMCs show comparable differentiation, but different proliferation behavior. **(A)** FACS analysis of wt, Lyn-/-, SHIP1-/-, and dko BMMCs showing comparable double-positivity of IgE-preloaded living BMMCs stained against Kit (anti-Kit-PE) and FcεRI-IgE-complex (anti-IgE-FITC). **(B)** Surface expression of ST2 and CD13 of living cells either unstained (grey line) or stained against ST2 (anti-ST2-FITC) and CD13 (anti-CD13-PE) (black line) was detected by flow cytometry. **(C)** Whole-cell lysates of untreated BMMCs were subjected to WB analysis with antibodies against SHIP1 (top panel), Lyn (middle panel), and p85 (loading control, bottom panel). **(D)** BMMCs were plated at 2x10^4^ cells/well in growth medium and the viable cell numbers were determined with a Casy cell counter after 4, 7, and 10 days. Each value is the mean of triplicates ± SD. The asterisks correspond to the differences between dko and wt, SHIP1-/- and wt as well as Lyn-/- and wt BMMCs; only after 4 days of culture the difference between Lyn-/- and wt cells was not significant. Comparable results were obtained with cells from different BMMC cultures (*n* > 3 (A, C); *n* = 3 (B); *n* = 2 (D)). (PDF 1615 kb) Additional file 3: Figure S3. Increased production of proinflammatory cytokines in Lyn- and SHIP1-deficient mast cells. Wt, Lyn-/-, SHIP1-/-, and dko BMMCs were preloaded and starved overnight with 0.15 μg/ml IgE. **(A, B)** BMMCs were left unstimulated (con) or stimulated with the indicated concentrations of Ag (ng/ml) for 4 h. Secreted IL-6 **(A)** and TNF-α **(B)** were measured by ELISA. Each bar is the mean of triplicates ± SD. **(C, D)** BMMCs were left unstimulated (con) or stimulated with the indicated concentrations of Ag (ng/ml) for 90 min. The amounts of *Il6* mRNA **(C)** and *Tnf* mRNA **(D)** were determined by RT-qPCR using the Pfaffl method. Comparable results were obtained with cells from different BMMC cultures (*n* > 3 (A-D)). (PDF 176 kb) Additional file 4: Figure S4. Comparison of proinflammatory cytokine mRNA production in optimally Ag-stimulated wt, Lyn-/-, SHIP1-/-, and dko BMMCs. Wt, Lyn-/-, SHIP1-/-, and dko BMMCs were preloaded and starved overnight with 0.15 μg/ml IgE. BMMCs were left unstimulated (con) or stimulated with 20 ng/ml of Ag for 90 min. The amounts of *Il6* mRNA **(A)** and *Tnf* mRNA **(B)** were determined by RT-qPCR using the Pfaffl method. Data obtained by analyzing cells from independent cultures are shown (the results already shown in Figs. 2c & d are indicated). (PDF 296 kb) Additional file 5: Figure S5. Lyn-/- and dko BMMCs show compromised degranulation and initial Ca^2+^ mobilization in response to Ag triggering. **(A)** Wt, Lyn-/-, SHIP1-/-, and dko BMMCs were preloaded and starved overnight with 0.15 μg/ml IgE. BMMCs were left untreated (con) or stimulated with the indicated concentrations of Ag (ng/ml) for 20 min. Degranulation was determined by β-hexosaminidase assay. Each point is the mean of triplicates ± SD. **(B)** Wt, Lyn-/-, SHIP1-/-, and dko BMMCs were preloaded and starved overnight with 0.15 μg/ml IgE. Ca^2+^ mobilization was measured for 6 min by flow cytometry as described in Additional file 1: Figure S1. Comparable results were obtained with cells from different BMMC cultures (*n* > 3 (A); *n* = 3 (B)). (PDF 370 kb) Additional file 6: Figure S7. Reduced NFκB-dependent gene transcription in response to Ag in Lyn-/- and dko BMMCs. Wt, Lyn-/-, SHIP1-/-, and dko BMMCs were left unstimulated (con) or stimulated with Ag (20 ng/ml) for 90 min. The amounts of *Nfkbia* mRNA (upper panel) and *Tnfaip3* mRNA (lower panel) were measured by RT-qPCR. A comparison of analyses of different independent cell cultures is depicted. The results shown in Fig. 4b are indicated. (PDF 241 kb) Additional file 7: Figure S6. Lyn/Ca^2+^/calcineurin dependence of NFκB activation in Ag-triggered mast cells. **(A)** Wt, Lyn-/-, SHIP1-/-, and dko BMMCs were left unstimulated (con) or stimulated with the indicated concentrations of Ag (ng/ml) for 90 min. The amounts of *Nfkbia* mRNA (upper panel) and *Tnfaip3* mRNA (lower panel) were measured by RT-qPCR. **(B)** Wt and SHIP1-/- BMMCs, pretreated with (+) or without (-) 1 mM EDTA for 1 min, were left unstimulated (-) or stimulated with Ag (20 ng/ml) for 5 min. Whole-cell lysates were subjected to WB analysis with antibodies against P-IκBα (top panel), IκBα (middle panel), and p85 (loading control, bottom panel). Wt **(C)** and SHIP1-/- BMMCs **(D)** were treated as in Fig. 4c & d and IκBα phosphorylation in response to Ag triggering (5 min) was densitometrically and statistically analyzed compared to loading control. Comparable results were obtained with cells from different BMMC cultures (*n* = 3 (A, C, D); *n* = 2 (B)). (PDF 273 kb) Additional file 8: Figure S8. Lyn/Ca^2+^/calcineurin-dependent signaling controls Ag-induced transcription of differential genes. Wt, Lyn-/-, SHIP1-/-, and dko BMMCs were left unstimulated (con) or stimulated with Ag (20 ng/ml) for 90 min. The amounts of *Tnfsf9* mRNA **(A)**, *Tnip3* mRNA **(B)**, *Il1a* mRNA **(C)**, *Il1b* mRNA **(D)**, and *Stx11* mRNA **(E)** were measured by RT-qPCR. A comparison of analyses of different independent cell cultures is depicted. The results shown in Fig. 5a are indicated. (PDF 534 kb) Additional file 9: Figure S9. Ag-triggered *Nfkbia* mRNA production is dependent on calcineurin activity. Wt BMMCs were preloaded and starved overnight with 0.15 μg/ml IgE. BMMCs, pretreated with vehicle (DMSO), 100 nM CsA, or 3 μM INCA-6 for 30 min, were stimulated with Ag (20 ng/ml) for 4 h **(A)** or 30 min **(B). (A)** Secreted TNF-α was measured by ELISA. Each bar is the mean of triplicates ± SD. **(B)** The amount of *Nfkbia* mRNA was measured by RT-qPCR. Comparable results were obtained with cells from different BMMC cultures (*n* = 2 (A); n = 3 (B)). (PDF 74 kb)

## Abbreviations

-/-: deficient
Ag: antigen
BMMC: bone marrow-derived mast cell
CsA: cyclosporine A
DAG: diacylglycerol
dko: double-deficient
FcεRI: high-affinity receptor for IgE
*Inpp5d*: gene name for murine SHIP1
IP: immunoprecipitation
MC: mast cell
*Nfkbia*: gene name for murine IκBα
SHIP1: SH2-containing inositol 5’-phosphatase 1
*Tnfaip3*: gene name for murine A20
WB: Western blotting
WM: wortmannin
Y-P: tyrosine phosphorylation
