# Supplementary figures and images for: Differential Lyn-dependence of the SHIP1-deficient mast cell phenotype
Source: Cell Commun Signal. 2016 May 20;14:12. doi: 10.1186/s12964-016-0135-0 (PMC4874025; doi:10.1186/s12964-016-0135-0)

## Differential Lyn-dependence of the SHIP1-deficient mast cell phenotype

### Suppl. Figure 1

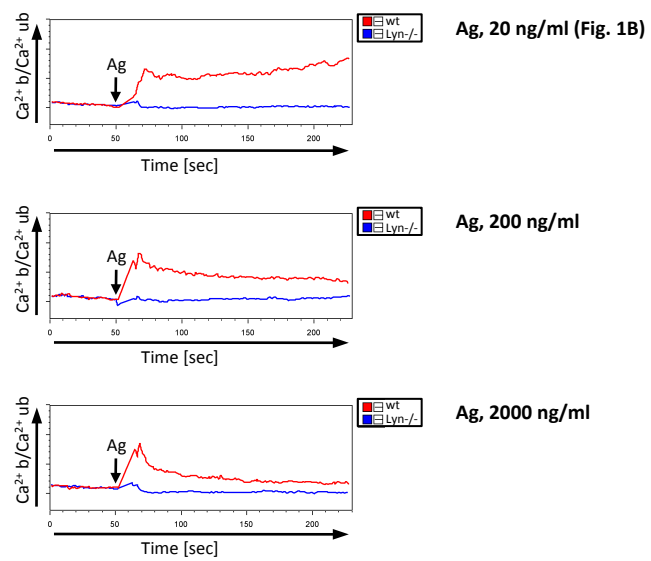

Supplement: Additional file 1: Figure S1. — Impaired Ca2+ mobilization in Lyn-/- BMMCs is independent of the stimulus concentration. Wt and Lyn-/- BMMCs were preloaded and starved overnight with 0.15 μg/ml IgE. Ca2+ mobilization was measured for 4 min by flow cytometry using the Ca2+-sensitive fluorescent dyes fluo-3 (Ca2+ b) and fura red (Ca2+ ub). Steady-state fluorescence was assessed for 1 min before BMMCs were stimulated with the indicated Ag concentrations. The arrow marks the time point of stimulus addition. Comparable results were obtained with cells from different BMMC cultures (n = 3). (PDF 289 kb) [file 12964_2016_135_MOESM1_ESM.pdf]

# Differential Lyn-dependence of the SHIP1-deficient mast cell phenotype

## Suppl. Figure 2

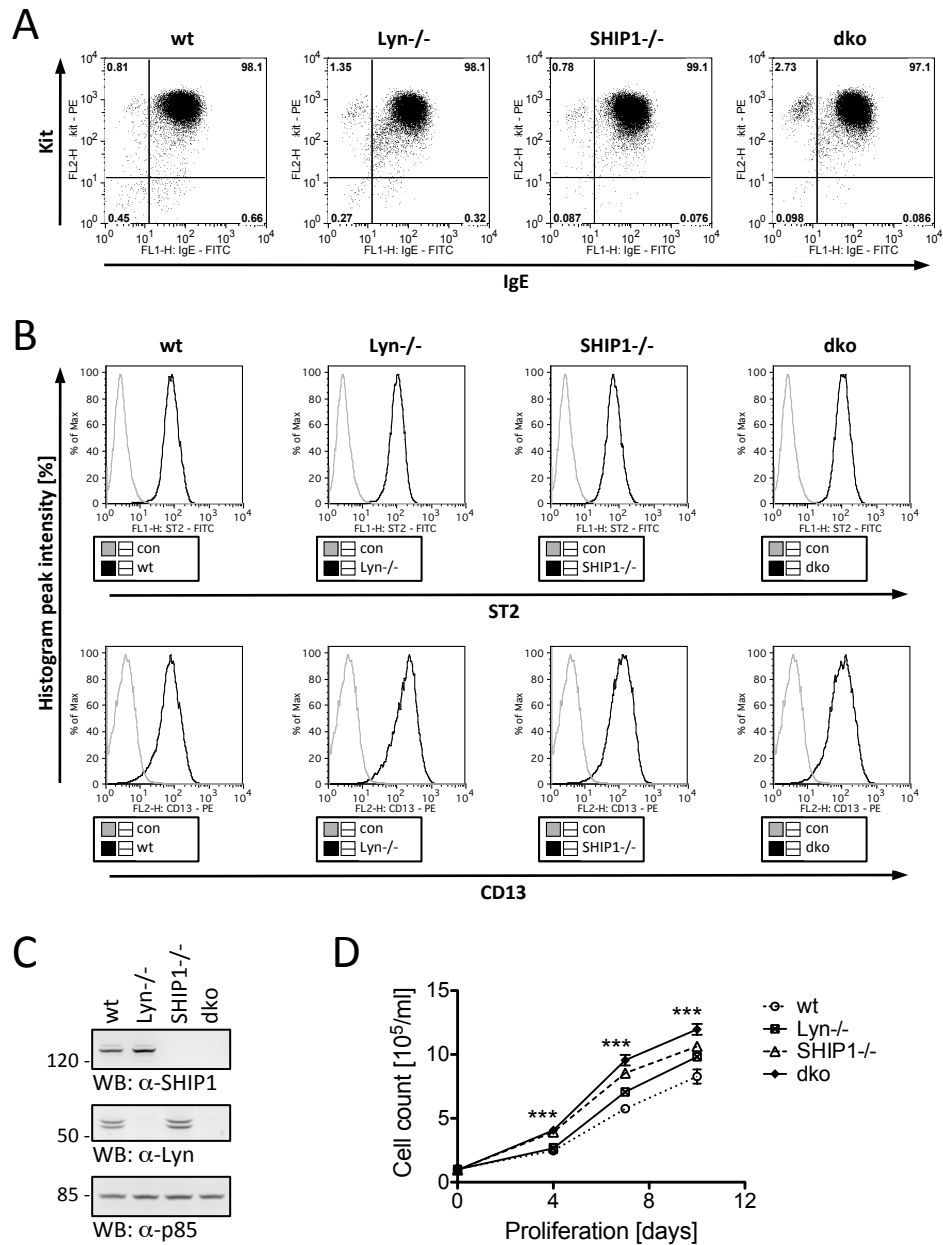

Supplement: Additional file 2: Figure S2. — Wt, Lyn-/-, SHIP1-/- and dko BMMCs show comparable differentiation, but different proliferation behavior. (A) FACS analysis of wt, Lyn-/-, SHIP1-/-, and dko BMMCs showing comparable double-positivity of IgE-preloaded living BMMCs stained against Kit (anti-Kit-PE) and FcεRI-IgE-complex (anti-IgE-FITC). (B) Surface expression of ST2 and CD13 of living cells either unstained (grey line) or stained against ST2 (anti-ST2-FITC) and CD13 (anti-CD13-PE) (black line) was detected by flow cytometry. (C) Whole-cell lysates of untreated BMMCs were subjected to WB analysis with antibodies against SHIP1 (top panel), Lyn (middle panel), and p85 (loading control, bottom panel). (D) BMMCs were plated at 2x10^4^ cells/well in growth medium and the viable cell numbers were determined with a Casy cell counter after 4, 7, and 10 days. Each value is the mean of triplicates ± SD. The asterisks correspond to the differences between dko and wt, SHIP1-/- and wt as well as Lyn-/- and wt BMMCs; only after 4 days of culture the difference between Lyn-/- and wt cells was not significant. Comparable results were obtained with cells from different BMMC cultures (n > 3 (A, C); n = 3 (B); n = 2 (D)). (PDF 1615 kb) [file 12964_2016_135_MOESM2_ESM.pdf]

## Differential Lyn-dependence of the SHIP1-deficient mast cell phenotype

Suppl. Figure 3

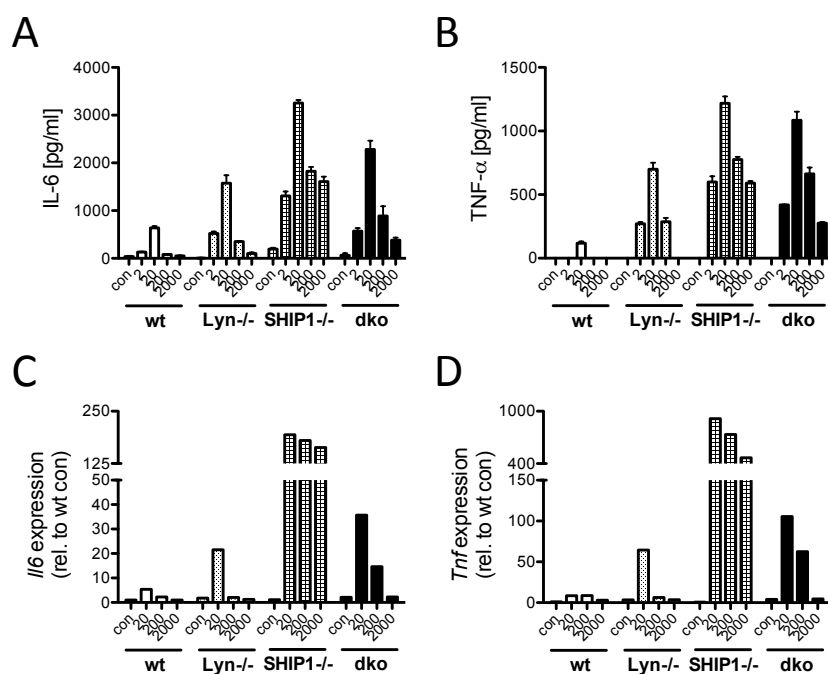

Supplement: Additional file 3: Figure S3. — Increased production of proinflammatory cytokines in Lyn- and SHIP1-deficient mast cells. Wt, Lyn-/-, SHIP1-/-, and dko BMMCs were preloaded and starved overnight with 0.15 μg/ml IgE. (A, B) BMMCs were left unstimulated (con) or stimulated with the indicated concentrations of Ag (ng/ml) for 4 h. Secreted IL-6 (A) and TNF-α (B) were measured by ELISA. Each bar is the mean of triplicates ± SD. (C, D) BMMCs were left unstimulated (con) or stimulated with the indicated concentrations of Ag (ng/ml) for 90 min. The amounts of Il6 mRNA (C) and Tnf mRNA (D) were determined by RT-qPCR using the Pfaffl method. Comparable results were obtained with cells from different BMMC cultures (n > 3 (A-D)). (PDF 176 kb) [file 12964_2016_135_MOESM3_ESM.pdf]

## Suppl. Figure 4

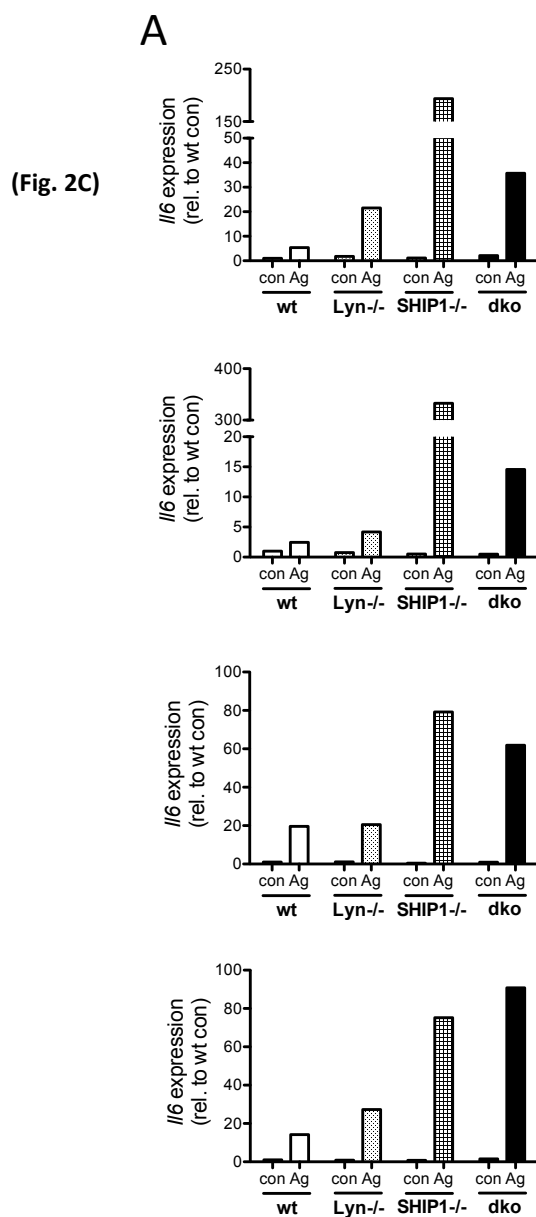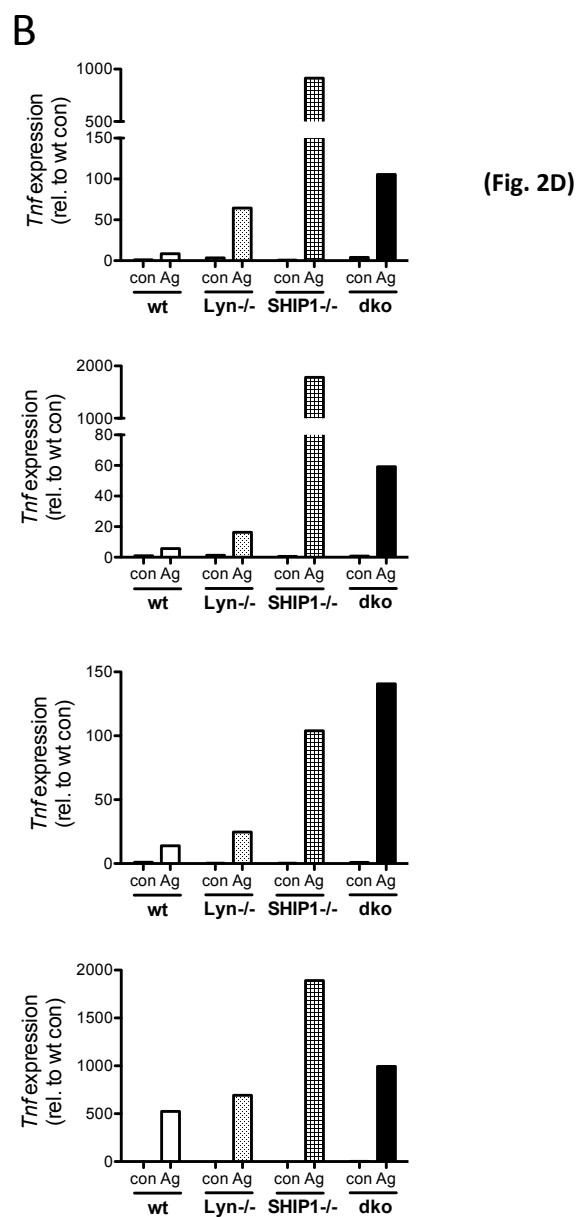

Supplement: Additional file 4: Figure S4. — Comparison of proinflammatory cytokine mRNA production in optimally Ag-stimulated wt, Lyn-/-, SHIP1-/-, and dko BMMCs. Wt, Lyn-/-, SHIP1-/-, and dko BMMCs were preloaded and starved overnight with 0.15 μg/ml IgE. BMMCs were left unstimulated (con) or stimulated with 20 ng/ml of Ag for 90 min. The amounts of Il6 mRNA (A) and Tnf mRNA (B) were determined by RT-qPCR using the Pfaffl method. Data obtained by analyzing cells from independent cultures are shown (the results already shown in Figs. 2c & d are indicated). (PDF 296 kb) [file 12964_2016_135_MOESM4_ESM.pdf]

## Differential Lyn-dependence of the SHIP1-deficient mast cell phenotype

### Suppl. Figure 5

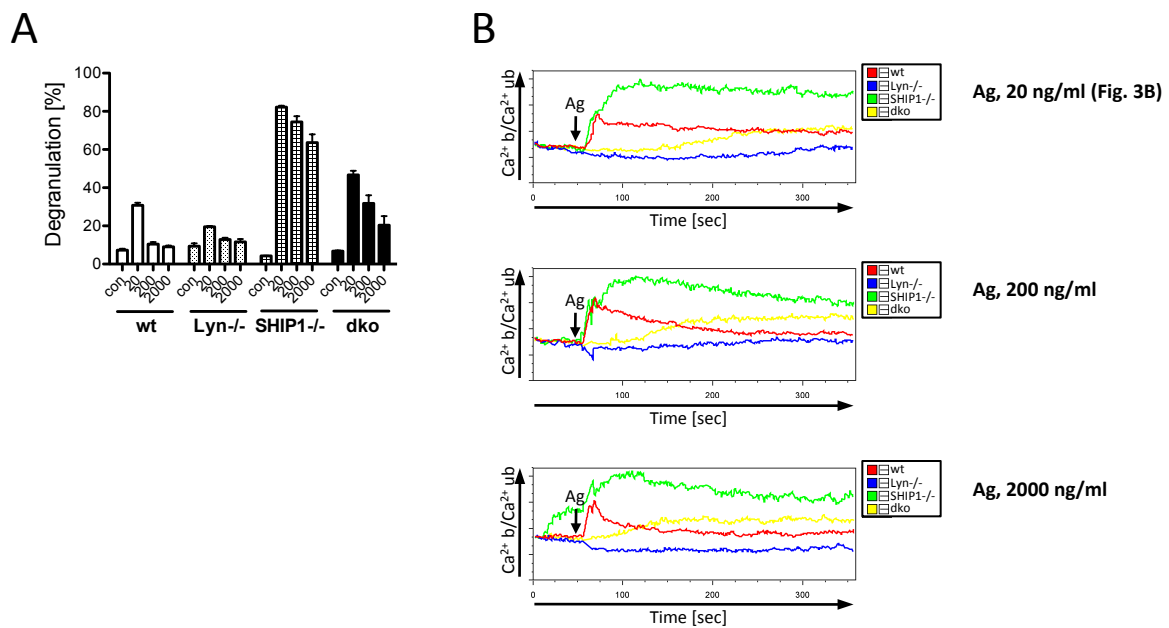

Supplement: Additional file 5: Figure S5. — Lyn-/- and dko BMMCs show compromised degranulation and initial Ca2+ mobilization in response to Ag triggering. (A) Wt, Lyn-/-, SHIP1-/-, and dko BMMCs were preloaded and starved overnight with 0.15 μg/ml IgE. BMMCs were left untreated (con) or stimulated with the indicated concentrations of Ag (ng/ml) for 20 min. Degranulation was determined by β-hexosaminidase assay. Each point is the mean of triplicates ± SD. (B) Wt, Lyn-/-, SHIP1-/-, and dko BMMCs were preloaded and starved overnight with 0.15 μg/ml IgE. Ca2+ mobilization was measured for 6 min by flow cytometry as described in Additional file 1: Figure S1. Comparable results were obtained with cells from different BMMC cultures (n > 3 (A); n = 3 (B)). (PDF 370 kb) [file 12964_2016_135_MOESM5_ESM.pdf]

## Differential Lyn-dependence of the SHIP1-deficient mast cell phenotype

### Suppl. Figure 7

(Fig. 4B)

A

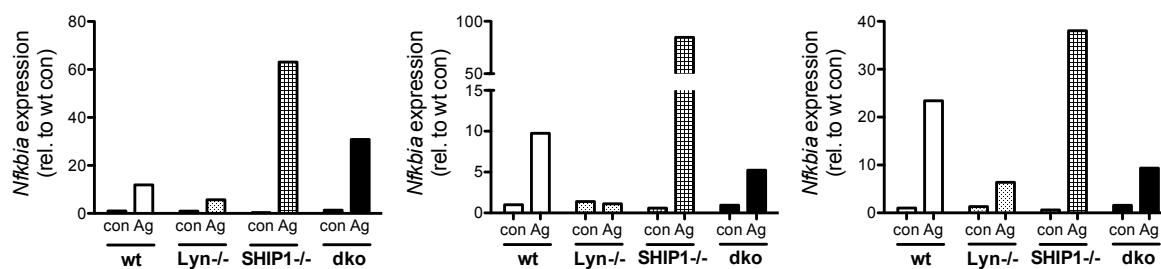

B

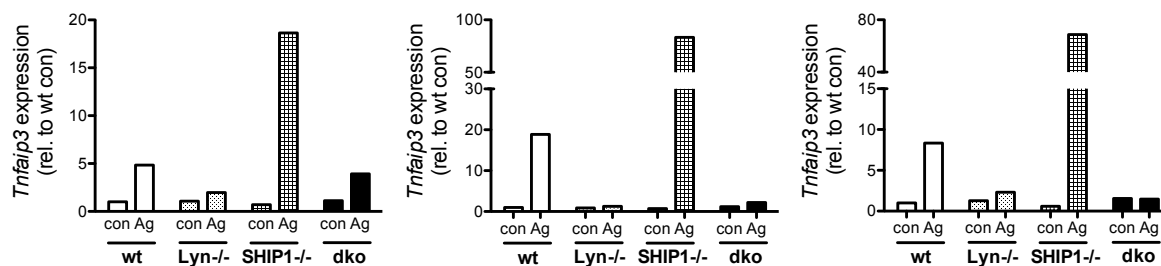

Supplement: Additional file 6: Figure S7. — Reduced NFκB-dependent gene transcription in response to Ag in Lyn-/- and dko BMMCs. Wt, Lyn-/-, SHIP1-/-, and dko BMMCs were left unstimulated (con) or stimulated with Ag (20 ng/ml) for 90 min. The amounts of Nfkbia mRNA (upper panel) and Tnfaip3 mRNA (lower panel) were measured by RT-qPCR. A comparison of analyses of different independent cell cultures is depicted. The results shown in Fig. 4b are indicated. (PDF 241 kb) [file 12964_2016_135_MOESM6_ESM.pdf]

# Differential Lyn-dependence of the SHIP1-deficient mast cell phenotype

## Suppl. Figure 6

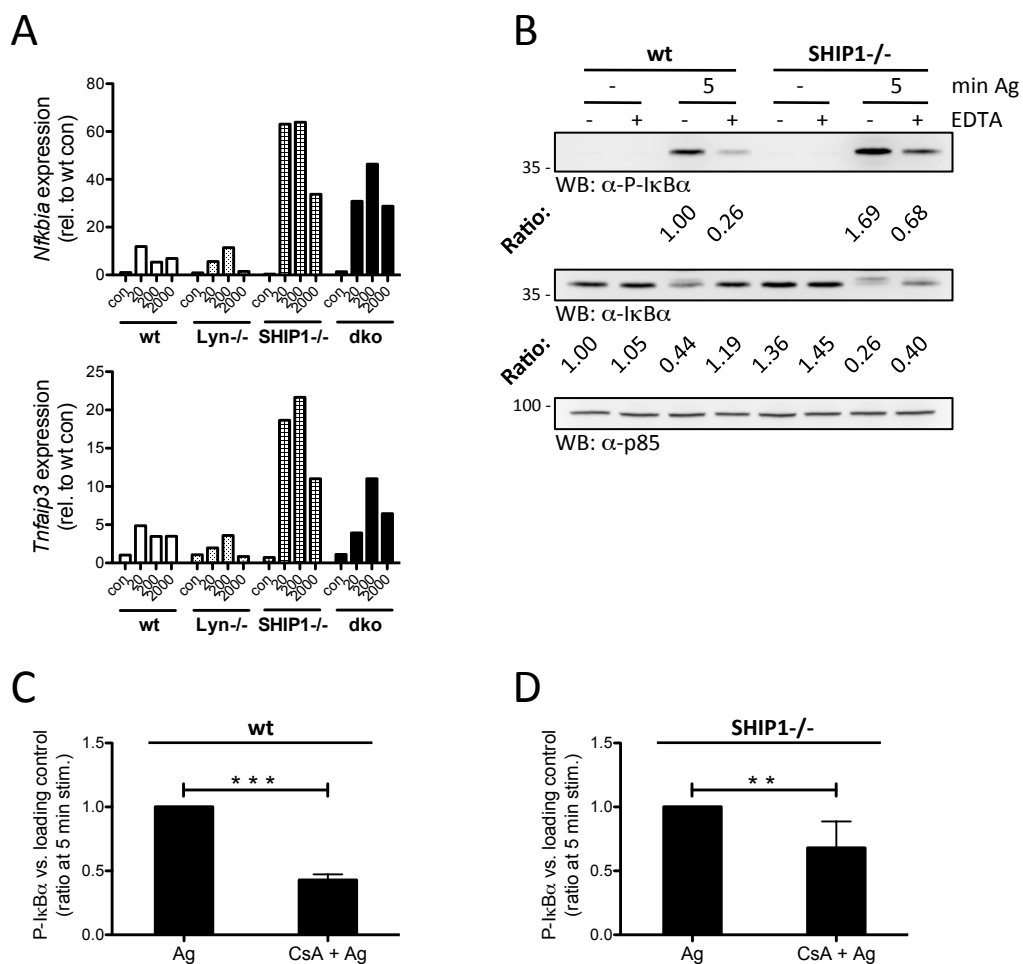

Supplement: Additional file 7: Figure S6. — Lyn/Ca2+/calcineurin dependence of NFκB activation in Ag-triggered mast cells. (A) Wt, Lyn-/-, SHIP1-/-, and dko BMMCs were left unstimulated (con) or stimulated with the indicated concentrations of Ag (ng/ml) for 90 min. The amounts of Nfkbia mRNA (upper panel) and Tnfaip3 mRNA (lower panel) were measured by RT-qPCR. (B) Wt and SHIP1-/- BMMCs, pretreated with (+) or without (-) 1 mM EDTA for 1 min, were left unstimulated (-) or stimulated with Ag (20 ng/ml) for 5 min. Whole-cell lysates were subjected to WB analysis with antibodies against P-IκBα (top panel), IκBα (middle panel), and p85 (loading control, bottom panel). Wt (C) and SHIP1-/- BMMCs (D) were treated as in Fig. 4c & d and IκBα phosphorylation in response to Ag triggering (5 min) was densitometrically and statistically analyzed compared to loading control. Comparable results were obtained with cells from different BMMC cultures (n = 3 (A, C, D); n = 2 (B)). (PDF 273 kb) [file 12964_2016_135_MOESM7_ESM.pdf]

## Differential Lyn-dependence of the SHIP1-deficient mast cell phenotype

### Suppl. Figure 9

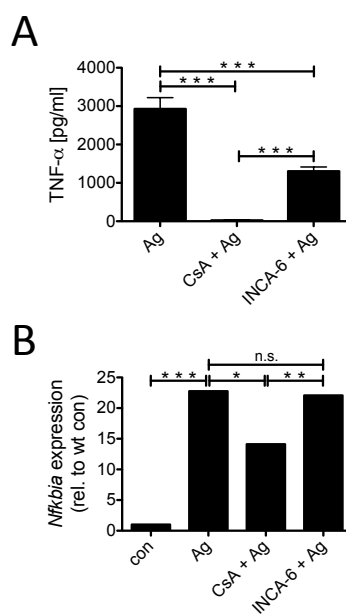

Supplement: Additional file 9: Figure S9. — Ag-triggered Nfkbia mRNA production is dependent on calcineurin activity. Wt BMMCs were preloaded and starved overnight with 0.15 μg/ml IgE. BMMCs, pretreated with vehicle (DMSO), 100 nM CsA, or 3 μM INCA-6 for 30 min, were stimulated with Ag (20 ng/ml) for 4 h (A) or 30 min (B). (A) Secreted TNF-α was measured by ELISA. Each bar is the mean of triplicates ± SD. (B) The amount of Nfkbia mRNA was measured by RT-qPCR. Comparable results were obtained with cells from different BMMC cultures (n = 2 (A); n = 3 (B)). (PDF 74 kb) [file 12964_2016_135_MOESM9_ESM.pdf]
