# Supplementary material for: Differential Lyn-dependence of the SHIP1-deficient mast cell phenotype
Source: Cell Commun Signal. 2016 May 20;14:12. doi: 10.1186/s12964-016-0135-0 (PMC4874025; doi:10.1186/s12964-016-0135-0)
Supplement: Additional file 8: Figure S8. — Lyn/Ca2+/calcineurin-dependent signaling controls Ag-induced transcription of differential genes. Wt, Lyn-/-, SHIP1-/-, and dko BMMCs were left unstimulated (con) or stimulated with Ag (20 ng/ml) for 90 min. The amounts of Tnfsf9 mRNA (A), Tnip3 mRNA (B), Il1a mRNA (C), Il1b mRNA (D), and Stx11 mRNA (E) were measured by RT-qPCR. A comparison of analyses of different independent cell cultures is depicted. The results shown in Fig. 5a are indicated. (PDF 534 kb) [file 12964_2016_135_MOESM8_ESM.pdf]

Differential Lyn-dependence of the SHIP1-deficient mast cell phenotype

Suppl. Figure 8

(Fig. 5A)

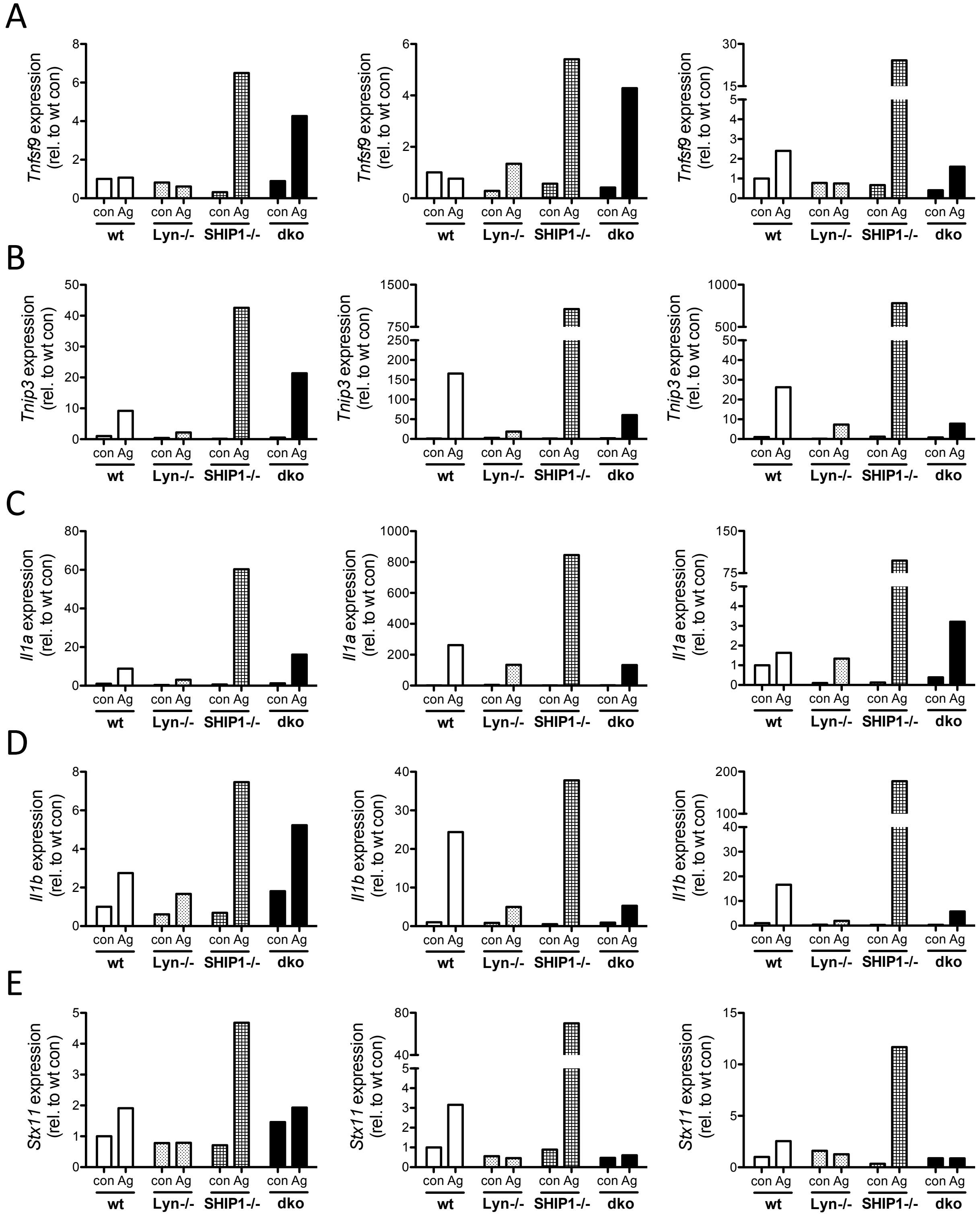

Nunes de Miranda *et al.* 2016
